# EHMTI-0156. Quantitative sensory testing in patients with headache attributed by idiopathic intracranial hypertension – a case-control study

**DOI:** 10.1186/1129-2377-15-S1-C65

**Published:** 2014-09-18

**Authors:** H Yri, S Munksgaard, L Bendtsen, R Jensen

**Affiliations:** 1 Danish Headache Center, Neurology, Copenhagen, Denmark

## Background

In patients with idiopathic intracranial hypertension (IIH) headache often persist as a disabling symptom even after intracranial pressure (ICP) has normalized. Yet very little is known about the mechanisms of chronification.

## Aim

To explore pain perception in patients with IIH in a controlled design at time of diagnosis and after 3 months of treatment.

## Materials and methods

We explored pain perception in patients with newly diagnosed IIH by Quantitative Sensory Testing (QST) measuring cephalic and extra-cephalic supra-threshold pain ratings and pain thresholds for pressure and electrical stimulation. QST was performed at diagnosis and after one and three months. ICP was measured at baseline and at the 3-month follow-up. QST measurements from sex-matched controls were used for comparisons. Headache was assessed by monthly standardized interviews and headache diaries.

## Results

At baseline IIH patients (n=28) showed no consistent abnormalities in pain sensitivity or thresholds (p>0.09 for all comparisons to healthy controls (n=28). Although headache improved markedly and ICP normalized in 52%, there was no consistent change in pain sensitivity from baseline to follow-up (p>0.09 for all variables). Patients with (54%) and without persistent chronic headache (46%) 3 months after diagnosis showed no different pain perception either at baseline or at the 3-month follow-up.

## Conclusions

Although headache persisted as a chronic symptom in half of the patients we found no evidence of increased central pain sensitivity suggesting that headache chronification in IIH is caused by mechanisms other than central sensitization.

